# Lymph node metastasis‐dependent molecular classification in papillary thyroid carcinoma defines aggressive metastatic outgrowth

**DOI:** 10.1002/ctm2.1211

**Published:** 2023-03-16

**Authors:** Dong Hyun Seo, Seul Gi Lee, Hwa Young Lee, Seonhyang Jeong, Sunmi Park, Jandee Lee, Young Suk Jo

**Affiliations:** ^1^ Department of Internal Medicine Yonsei University College of Medicine Seoul South Korea; ^2^ Department of Surgery Daejeon Eulji Medical Center Eulji University Daejeon South Korea; ^3^ Department of Surgery Open NBI Convergence Technology Research Laboratory Severance Hospital Yonsei Cancer Center Yonsei University College of Medicine Seoul South Korea

Dear Editor,

Lymph node metastasis (LNM) is the most important prognostic factor and a crucial indicator in the development of treatment strategies for patients with papillary thyroid carcinoma (PTC).^1^, ^2^ A vast majority (25%–60%) of patients with PTC undergo thyroidectomy with neck node dissection.^3^ However, researchers continue to debate whether LNM is leading to over‐treatment of patients with PTC.^1^, ^4^ Interestingly, we have noted that upon stratifying LNM risk in PTC and clinical outcomes according to distinct molecular characteristics, LNM showing enrichment of genes related to inflammation and epithelial–mesenchymal transition (EMT) were more aggressive than metabolically adapted LNM, and this was applicable in other tumours.

A flowchart of this study is displayed in Figure S1. For discovery, we ran differential gene expression analysis based on the negative binomial distribution (DESEQ2) for 97 patients without LNM [LNM (−)] and 195 patients with LNM [LNM (+)] from our PTC patient cohort (Table S1). One hundred four differentially expressed genes (DEGs) were upregulated in LNM (+), whereas the other 140 DEGs were downregulated (Figure 1A). In LNM (+) specimens, DAVID software revealed significant clustering of up‐regulated genes related with EMT pathways (Figure S2A–C). Subsequent analysis of DEGs using the *K*‐means unsupervised clustering algorithm was performed to define marked molecular subtypes, wherein *K* = 2 showed the greatest classification outcome with a cophenetic coefficient value of.736 (Figure 1B, Figures S3 and S4). To develop a predictive scoring model, we employed LASSO regression and 18 gene signatures that effectively deciphered molecular subtypes with an area under curve value of .97 (Figure 1C–E, Figures S5A–C and S6A,B).

**FIGURE 1:**
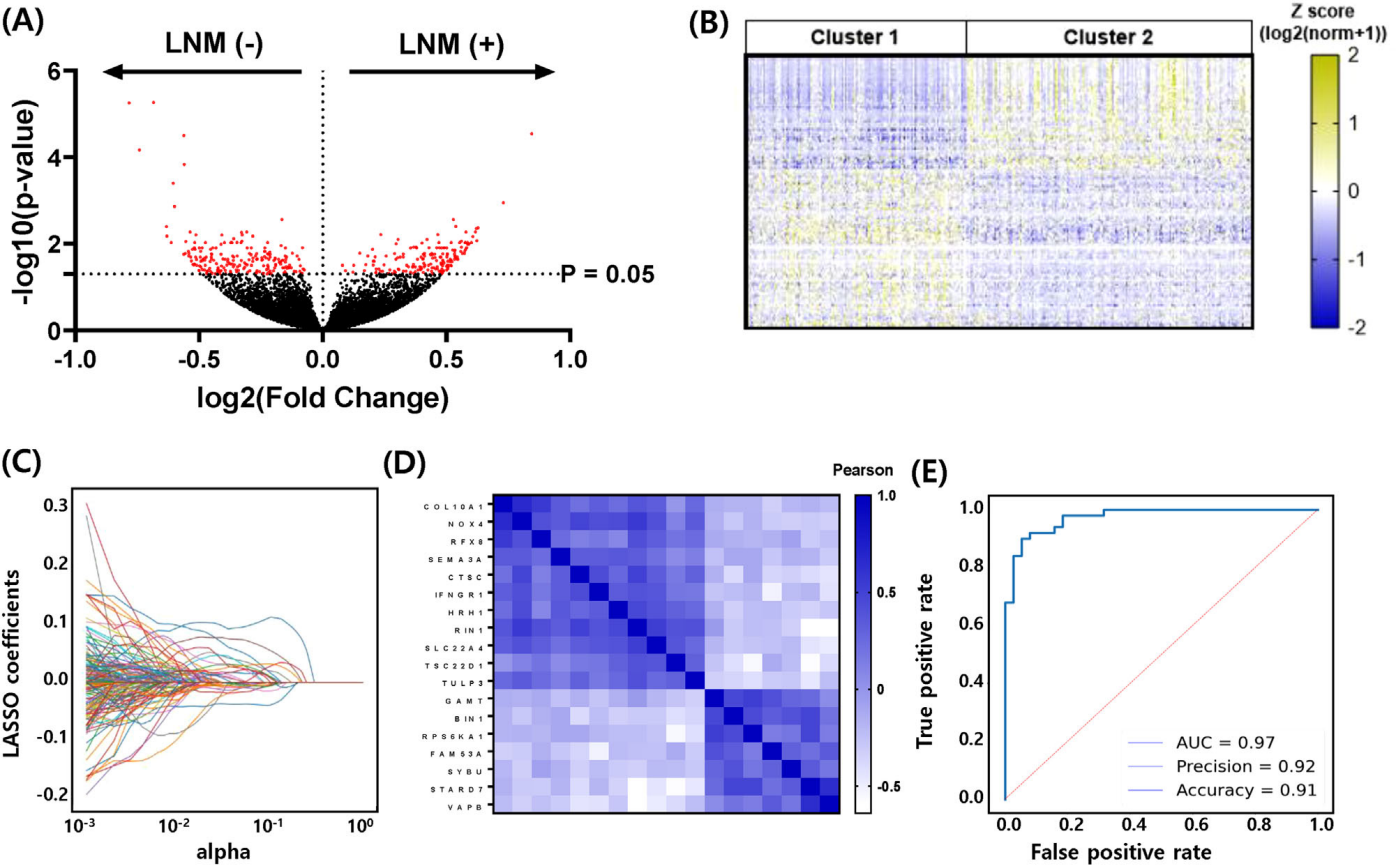
Discovery of novel molecular subtypes and machine learning model construction. (A) Volcano plot of DESEQ2 in our in‐house PTC patient cohort shows differential transcriptional patterns. The dotted line was drawn to demarcate the threshold of DEGs with a *p*‐value of.05 (−log10(*p*‐value) of 1.301). The *X*‐axis represents fold changes in log2 scale, which was calculated as log2(average LNM (+) gene expression/average LNM (−) gene expression). Additionally, 244 significant DEGs (Bonferroni adjusted *p*‐value < .05) are indicated in red. (B) Heatmap of two distinctive molecular subtypes in our in‐house PTC patient cohort were found via *K*‐means clustering. Expressional levels of candidate genes are presented as *z*‐scores of log 2 (normalised count + 1). (C) Determination of gene signatures by LASSO regularisation analysis. A dotted line was drawn to represent 95% confidence intervals of cross validation scores. (D) Heatmap of Pearson correlation coefficients tested against expression profiles of every 18 gene signature. (E) Receiver operating characteristic curve of the test data set. BP, biological process; CC, cellular component; DAVID, database for annotation, visualisation, and integrated discovery; DEGs, differentially expressed genes; DESEQ2 differential gene expression analysis based on the negative binomial distribution; GO, gene ontology; MF, molecular function;; LNM, lymph node metastasis; PTC, papillary thyroid cancer.

We then characterised the molecular characteristics of differently defined clusters by evaluating hallmark gene sets of a molecular signature database using single sample gene set enrichment analysis (ssGSEA).^5^ PTC patients from Cluster 1 in both our cohort and TCGA shared up‐regulated patterns of metabolism‐related hallmark pathways. PTC patients from Cluster 2 in both our cohort and TCGA exhibited common enrichment of EMT and immune response (Figure 2A, Figure S7). These differences in enrichment were not noted when patient samples were compared according to LNM status (Figure S8A,B).

**FIGURE 2:**
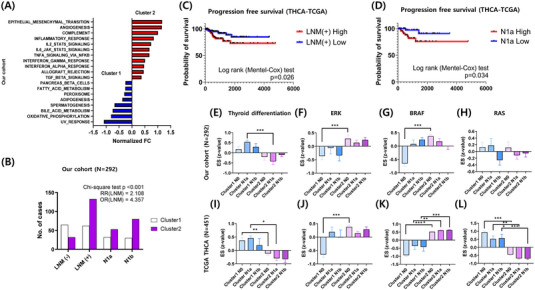
Characterisation of novel molecular subtypes of PTC. (A) Bar graph of hallmark gene set enrichment scores compared between Cluster 1 and Cluster 2 of PTC patients in our cohort. Hallmark gene sets significantly enriched with *p*‐values below.01 are presented. (B) Number of cases LNM (−), LNM (+), N1a, and N1b specifically depicted as a bar graph in each cluster. Cluster 2 and Cluster 1 have a correlated LNM risk, odds ratio of 4.357 (95% CI: 2.622–7.295) in our cohort. (C, D) Progression free survival according to high and low LASSO scores in patients with LNM (+) PTC. N1a was compared. Log rank (Mantel–Cox) test was performed to prove the significance of Kaplan–Meier survival curves. ssGSEA enrichment scores for (E, I) thyroid differentiation, (F, J) ERK pathway, (G, K) BRAF, and (H, L) RAS are plotted. Significance bars are only drawn for tumour groups with the same LNM status in each cluster. Error bars are presented with standard errors of the mean. *p < .05, **p < .01, ***p < .001. OR, odds ratio; RR, relative risk; LNM, lymph node metastasis; PTC, papillary thyroid cancer.

We noticed a dominant proportion of LNM (+) patients (133 [80.6%]) in Cluster 2 in our cohort. Remarkably, 80 N1b (cancer with lateral neck node metastasis) patient samples (48.5%) were allocated in Cluster 2, while 32 (25.2%) N1a (cancer with central neck node metastasis) and 30 (23.6%) N1b patient samples were in Cluster 1. The odds ratio to observe LNM in Cluster 2 was 4.098 (Figure 2B). Validation with an odds ratio of 4.691 was observed for LNM in Cluster 2, which indicates a strong clinical association between Cluster 2 and LNM (Figure S9). We then set out to determine the prognostic significance of our signature genes related with disease progression or recurrence by integrating clinical survival data from the TCGA database. In doing so, we noted that patients with LNM and high LASSO scores in Cluster 2 (upper two thirds of the designated cluster) displayed significant differences in their prognosis, compared to those with low LASSO scores (lower two‐thirds of the designated cluster) among LNM patients in Cluster 1 (Figure 2C). Interestingly, aggressive outcomes were also noticed in N1a patients from Cluster 2 (Figure 2D). This is a crucial issue, as it is not clear whether pathological N1a metastasis can serve as a risk factor in patients with PTC.^6^ Furthermore, we evaluated thyroid differentiation scores and other important PTC‐related oncogenic pathways and found corroborating evidence of biological aggressiveness in Cluster 2 (Figure 2E–L, Table S2). Meanwhile, progression‐free survival according to LNM status or the presence of BRAF mutation, a unique genomic marker of PTC, did not show prognostic significance (Figure S10A,B).

Additionally, we compared demographic and genomic information from PTC patients between different molecular clusters, as well as multivariate linear regression with LASSO score, to seek for any potential clinical relevance (Tables 1 and S3). Notably, BRAF^V600E^ mutation, T stage, degree of LNM, and extrathyroidal extension (ETE) differed significantly between two clusters, and depth of ETE exhibited a significant proportionate relationship with LASSO score, suggesting a greater chance of severe metastasis in Cluster 2 PTC patients (Tables S4–S6).

**TABLE 1:** Multivariable linear regression analysis of other clinical factors and LASSO scores in our cohort and the TCGA THCA dataset.

Risk score						
	Our cohort (*n* = 292)			TCGA (*n* = 451)		
	β*	*P*	95% CI	β*	*p*	95% CI
**Intercept**	.3446	<.001^***^	.203 to .486	.6111	<.001^***^	.492 to .731
**Age**	−.000	.333	−.003 to .007	−0.001	.120	−.003 to 0
**Gender**						
Female	[Reference]					
Male	−.033	.203	−.101 to .034	−.013	.644	−.0690 to .043
**Extrathyroidal extension**						
None	[Reference]					
Minimal (Yes)	.1770	<.001^***^	.104 to .249	.199	<.001^***^	.113 to .286
Moderate/advanced			.406	.002^**^	.147 to .666	
Very advanced			.716	.01*	.173 to 1.269	
**T stage**						
T1	[Reference]					
T2	−.096	.296	−.278 to .085	.073	.096	−.026 to .204
T3	.156	<.001^***^	.079 to .234	.089	.129	−.013 to .16
T4	.171	.003**	.058 to .285	.016	.895	−.223 to .255
**Other thyroid disease**						
Normal	[Reference]					
Lymphocytic thyroiditis	–	–	–	−.009	.899	−.143 to .126
Other, specify	–	–	–	−.075	.975	−.227 to .076

* β coefficients represent the effect of moving from the “Reference” category. β coefficients of reference categories are set to zero because it is redundant. CI, confidence interval; TCGA, the cancer genome atlas; THCA, thyroid cancer.

Our gene expression program was extended to actual LNM samples of PTC by applying it to public databases, GSE60542 and GSE151179 (Figure 3A, S11A–F).^7^, ^8^ Also, we challenged the applicability of our gene expression program in LNM of other tumour types and incorporated datasets of breast cancer and melanoma from TCGA, GSE56493, and GSE65904.^9^, ^10^ Likewise, molecular subtypes were deciphered using our proposed LASSO gene expression program, and identified molecular characteristics were validated in both primary tumour and LNM (Figure 3A). Moreover, LASSO scores of paired primary tumour and LNM samples showed significant relevance, suggesting preservation of molecular profiles after disease progression (Figure S12A–E). Analogous tendencies were observed in poorly differentiated thyroid carcinoma and anaplastic thyroid cancer patients in GSE76039 (Figure S13A,B).

**FIGURE 3:**
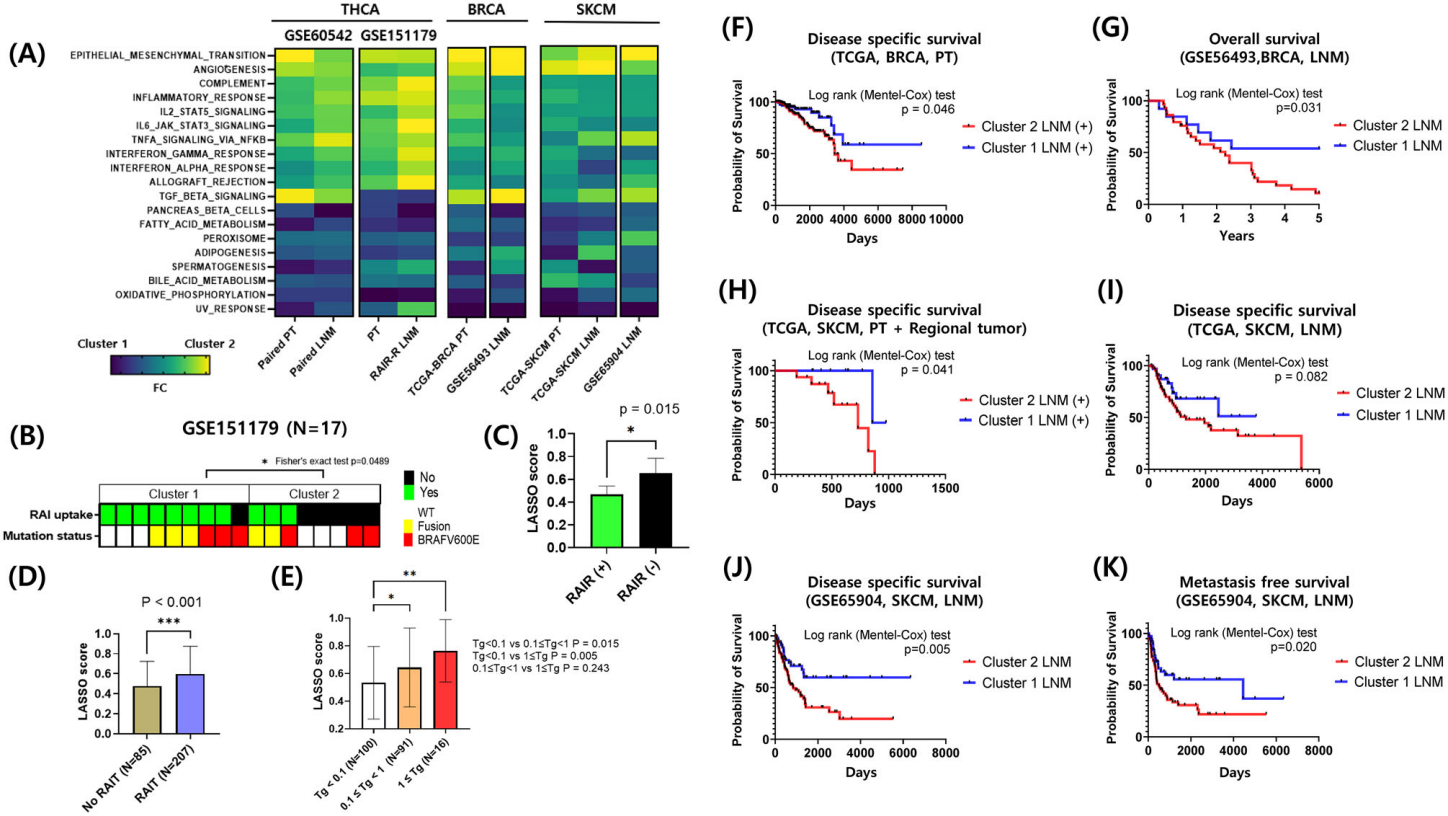
Validation of molecular subtypes in LNM samples and other cancer cohorts. (A) Results of ssGSEA analysis for GSE60542 paired primary tumour and LNM samples and GSE151179 primary tumour and LNM samples were figured into a heatmap. Results of enrichment analysis for TCGA‐BRCA, GSE56493, TCGA‐SKCM, and GSE65904 were enrolled for comparison. Genes are listed in the order of significance evaluated in our discovery cohort. Fold change was calculated as a comparison of average enrichment scores in each molecular cluster. (B) Panels representing RAI uptake and mutational status of PTC samples in GSE151179 are depicted as molecular clusters. Fisher's exact test was performed to evaluate differences in the frequency of RAI uptake in both clusters. (C) LASSO scores were compared between patients in GSE151179 with different responses after RAIT. (D, E) LASSO scores were compared in our in‐house database of patients according to whether or not RAIT was performed and serum thyroglobulin levels 1 year after RAIT. Mann–Whitney *U*‐test was performed for statistical analysis. (F, H, I, J) Disease‐specific survival was compared between molecular subtypes in either PTC samples with LNM or LNM. Log rank (Mantel–Cox) test was performed to prove significance of Kaplan–Meier survival curves. (G) Overall survival rate was assessed between molecular subtypes of GSE56493 LNM samples. (K) Metastatic free survival was compared according to molecular subtypes from GSE65904 LNM samples. *P < .05, **P < .01, ***P < .001. BRCA, breast cancer; LNM, lymph node metastasis; PT, primary tumour; RAI, radioactive iodine treatment; RAI‐R, radioactive iodine treatment refractory; SKCM, skin cutaneous melanoma; Tg, thyroglobulin.

To expand the program's clinical significance, we analysed treatment responses to radioiodine therapy among PTC patients in GSE151179. Notably, significant radioactive iodine (RAI) uptake was found in Cluster 1 with markedly lower LASSO scores than patients without radioiodine uptake (Figure 3B,C). In addition, LASSO scores were higher in patients who underwent RAI treatment (RAIT) (Figure 3D) and in an intermediate‐ or high‐dose group, compared to no treatment or low‐dose group (Figure S14A, S14B). LASSO scores also showed a clear tendency to increase as suppressed Tg increased among all patients who underwent RAIT (Figure 3E) and patients who underwent high‐dose RAIT (Figure S14C). Finally, we observed concordance between the aggressive outcomes noticed in Cluster 2 primary tumours with LNM and LNM samples from other tumour types (Figure 3F–K).

Herein, we report two potential distinct LNM molecular subtypes of PTC with different clinical outcomes in terms of LNM and biological features. Proposed signatures were conserved throughout disease progression and validated in other thyroid cancer data and different tumour types. An EMT/inflammation axis, which we suggest as an aggressive LNM mechanism, requires support from additional studies for potential application in clinical settings.

## CONFLICTS OF INTERESTS STATEMENT

The authors declare that they have no competing interests.

## Supporting information

Supporting Information

## References

[1] Lee J , Kim CH , Min IK , et al. Detailed characterization of metastatic lymph nodes improves the prediction accuracy of currently used risk stratification systems in N1 stage papillary thyroid cancer. Eur J Endocrinol. 2020;183(1):83‐93. 32487777 10.1530/EJE-20-0131

[2] Lee WK , Lee J , Kim H , et al. Peripheral location and infiltrative margin predict invasive features of papillary thyroid microcarcinoma. Eur J Endocrinol. 2019;181(2):139‐149. 31146263 10.1530/EJE-18-1025

[3] Agrawal N , Akbani R , Aksoy BA , et al. Integrated genomic characterization of papillary thyroid carcinoma. Cell. 2014;159(3):676‐690. 25417114 10.1016/j.cell.2014.09.050 PMC4243044

[4] Cranshaw IM , Carnaille B . Micrometastases in thyroid cancer. An important finding? Surg Oncol. 2008;17(3):253‐258. 18504121 10.1016/j.suronc.2008.04.005

[5] Barbie DA , Tamayo P , Boehm JS , et al. Systematic RNA interference reveals that oncogenic KRAS‐driven cancers require TBK1. Nature. 2009;462(7269):108‐112. 19847166 10.1038/nature08460 PMC2783335

[6] Lee YM , Park JH , Cho JW , Hong SJ , Yoon JH . The definition of lymph node micrometastases in pathologic N1a papillary thyroid carcinoma should be revised. Surgery. 2019;165(3):652‐656. 30385127 10.1016/j.surg.2018.09.015

[7] Tarabichi M , Saiselet M , Trésallet C , et al. Revisiting the transcriptional analysis of primary tumours and associated nodal metastases with enhanced biological and statistical controls: application to thyroid cancer. Br J Cancer. 2015;112(10):1665‐1674. 25965298 10.1038/bjc.2014.665 PMC4430711

[8] Colombo C , Minna E , Gargiuli C , et al. The molecular and gene/miRNA expression profiles of radioiodine resistant papillary thyroid cancer. J Exp Clin Cancer Res. 2020;39(1):245. 33198784 10.1186/s13046-020-01757-x PMC7667839

[9] Tobin NP , Harrell JC , Lövrot J , et al. Molecular subtype and tumor characteristics of breast cancer metastases as assessed by gene expression significantly influence patient post‐relapse survival. Ann Oncol. 2015;26(1):81‐88. 25361981 10.1093/annonc/mdu498 PMC4269343

[10] Cirenajwis H , Ekedahl H , Lauss M , et al. Molecular stratification of metastatic melanoma using gene expression profiling: prediction of survival outcome and benefit from molecular targeted therapy. Oncotarget. 2015;6(14):12297‐12309. 25909218 10.18632/oncotarget.3655 PMC4494939

