# Supplementary material for: Lymph node metastasis‐dependent molecular classification in papillary thyroid carcinoma defines aggressive metastatic outgrowth
Source: Clin Transl Med. 2023 Mar 16;13(3):e1211. doi: 10.1002/ctm2.1211 (PMC10019769; doi:10.1002/ctm2.1211)
Supplement: Supplementary file 1 — Supporting Information [file CTM2-13-e1211-s001.docx]

Supplementary Information

**Lymph Node Metastasis Dependent Molecular Classification in Papillary Thyroid Carcinoma Defines Aggressive Metastatic Outgrowth.**

Dong Hyun Seo^1^, Seul Gi Lee^2^, Hwa Young Lee^3^, Seonhyang Jeong^1^, Sunmi Park^1^, Jandee Lee^3^* and Young Suk Jo^1^*

^1^Department of Internal Medicine, Yonsei University College of Medicine, Seoul, South Korea

^2^Department of Surgery, Daejeon Eulji Medical Center, Eulji University; Daejeon, South Korea

^3^Department of Surgery, Open NBI Convergence Technology Research Laboratory, Severance Hospital, Yonsei Cancer Center, Yonsei University College of Medicine, Seoul, South Korea

The first two authors equally contributed to this work.

Correspondence: jandee@yuhs.ac (J.L.) or joys@yuhs.ac (Y.S.J.)

This file includes:

Supplementary Materials and Methods

Tables S1‒S7

Figures S1‒S14

Supplementary References

**Supplementary Materials and Methods**

*Patients and specimen collection*

In this study, 292 patients with PTC who underwent thyroidectomy from May 2014 to January 2020 at Severance Hospital were enrolled. Of the 292 enrolled patients, 182 (62.3%) initially underwent thyroidectomy (TT) with prophylactic or therapeutic central neck node dissection (CCND), and 110 (37.7%) underwent TT with therapeutic modified radical neck dissection (MRND) for clinically suspicious and/or cytological confirmed lateral LNs. Therefore, the patients of this paper underwent at least CCND, and histological examination of LNM was sufficiently evaluated.^1^ Briefly, tissue samples were sectioned at 2-mm intervals following dissection from adipose tissue, after which the LN tissues were prepared for permanent section evaluation. For each LN, 4-5 μm thick sections were made at three levels from each piece submitted and stained with hematoxylin and eosin (H&E).^2-4^ Snap frozen tumour tissues were collected for nucleic acid extraction, and clinico-pathological information of patients was collected. All tissues were harvested and collected by a trained laboratory technician/technologist designated by the tumor biobank according to standard operating procedures (SOPs) as follows: 1) Cryovials were prepared as needed and labelled before starting the surgery. 2) After extracting the specimen from the patient, the specimen including the tumor was cut in half, and tissue was harvested from the central area of the tumor with clean scalpels and forceps between samples to avoid cross contamination. 3) The harvested tissue was placed into an empty cryovial, which was closed and immediately submerged into liquid nitrogen in a portable liquid nitrogen storage container. 4) The harvested samples and tissue harvesting time for each patient were recorded on the Tissue Collection/Harvesting Worksheet. 5) The samples were finally stored in a liquid nitrogen storage tank (preferred) or an -80º C (or colder) freezer, and the storage location was recorded. The average cold ischemic time for harvesting tissue was within 15 minutes from resection; this was measured from ligation of supplying vessels to thyroid tissue to snap freezing. To obtain accurate, precise, and reproducible data, quality control (QC) involved multiple steps. In the pre-operative step, we included patients with papillary thyroid cancer diagnosed by fine needle aspiration cytology (Bethesda System for Reporting Thyroid Cytopathology Category VI, Malignant). We also only included tumors >1.0 cm in size by ultrasonography for harvesting tissue to avoid affecting the final pathological diagnosis. In the operative step, we harvested the tissue sample from the central area of the tumor, and this was enough to prevent contamination with other non-tumor tissues. In the pre-analytic step, we excluded samples based on the following H&E pathology: 1) the absence of malignant cells or fewer than 100 malignant cells in the entire section, 2) mixed with another subtype of thyroid cancer, 3) tissue size smaller than 5 mm, and 4) fibrotic tissue without inflammatory cells and necrotic tissue. Written informed consent was obtained from all study participants, and this study was approved by the Institutional Review Board of Severance Hospital.

*Identification of BRAF, RAS, and TERT promoter mutations*

DNA extraction was performed using a QiaAmp DNA Mini kit (Qiagen Inc., Valencia, CA, USA) according to the manufacturer’s instructions. Genomic DNA was amplified by PCR using detailed primers (sequences are provided in Table S7) on a C1000 thermal cycler (Bio-Rad, Hercules, CA, USA). Distilled water and DNA from cell lines with known mutational status were used as negative and positive controls, respectively. Following electrophoresis on a 2% agarose gel, the product was visualised in the Gel Doc EZ system (Bio-Rad) and purified using QIAquick® Gel Extraction Kit (Qiagen Inc.). The sequencing was carried out by an ABI 3730XL DNA Analyser using a Big Dye terminator v3.1 cycle sequencing ready reaction kit (Applied Biosystems, Foster, CA, USA).

*Transcriptome analysis*

Total RNAs from tissues were extracted using TRIzol^Ⓡ^ (Invitrogen, Carlsbad, CA, USA). Total RNA concentration was calculated by Quant-IT RiboGreen (#R11490, Thermo Fisher Scientific, Hayward, CA, USA), and RNA integrity was assessed by TapeStation RNA screentape (#5067-5576, Agilent, Santa Clara, CA, USA). RNA preparations, with total amounts greater than or equal to 1.0 μg, RIN greater than or equal to 7.0, and rRNA ratio greater than or equal to 1.0 were used for RNA library construction. A library was independently prepared with 1 μg of total RNA for each sample using an Illumina TruSeq Stranded mRNA Sample Prep Kit (#RS-122-2101, Illumina, Inc., San Diego, CA, USA). First purifying of the poly‐A-containing mRNA molecules using poly‐T‐attached magnetic beads was performed. Next, the mRNA was fragmented into small pieces using divalent cations under elevated temperature. The cleaved RNA fragments were copied into first strand cDNA using SuperScript II reverse transcriptase (#18064014, Thermo Fisher Scientific) and random primers. This was followed by second strand cDNA synthesis using DNA Polymerase I, RNase H, and dUTP. These cDNA fragments underwent an end repair process, followed by the addition of a single ‘A’ base and ligation of the adapters. The products were then purified and enriched with PCR to create the final cDNA library. The libraries were quantified using KAPA Library Quantification kits for Illumina Sequencing platforms according to the qPCR Quantification Protocol Guide (#KK4854, KAPA BIOSYSTEMS, Wilmington, MA, USA) with the following primers: 5’-AAT GAT ACG GCG ACC ACC GAG AT-3’ (sense) and 5’- CAA GCA GAA GAC GGC ATA CGA-3’ (antisense). The quality of libraries was evaluated according to template size distribution using the TapeStation D1000 ScreenTape (# 5067-5582, Agilent Technologies, Santa Clara, CA, USA). Indexed libraries with concentrations greater than or equal to 10.0 nM and optimal size distributions were then submitted to an Illumina NovaSeq 6000 platform (Illumina Inc., San Diego, CA, USA), and paired-end (2 × 100 bp) sequencing was performed. After quality control using FastQC v0.11.7, the trimming of adaptors and low-quality bases with base quality less than 3 and sliding window trimming with a window size of 4 and a threshold quality score of Q15, with a read length filter retaining only reads longer than 36 bp in length, were performed using Trimmomatic 0.38. Trimmed reads were aligned to a reference genome GRCh37 (hg19) using HISAT2 v2.1.0 and assembled into transcripts using StringTie v2.1.3b.^5^ We extracted the expression profiles as read counts and fragments per kilobase of transcript per million mapped reads (FPKM) values considering transcript quantification, read counts, transcript length, and depth of coverage. Sequencing data were processed through g:Profiler, a web server for functional enrichment analysis.^6^

*Fusion gene analysis*

For the detection of fusion oncogenes, deFuse v0.8.1, FusionCatcher v1.00, and Arriba v.1.2.0 were applied to the expression data, and the results found simultaneously in all algorithms were selected as valid.^7-9^

*Quantitative real-time polymerase chain reaction (PCR)*

Total RNA was isolated from frozen tissues by the method used in transcriptome analysis, and RNA quality was assessed using a 2100 Bioanalyzer System (Agilent Technologies). Complementary DNA was generated from total RNA using the SuperScript™ III First-Strand Synthesis System according to the manufacturer’s protocol (Invitrogen, 18080051). Quantitative real-time PCR (qRT-PCR) was performed using a StepOne™ Real-Time PCR System (Applied Biosystems) with SYBR Green Supermix (Bio-Rad). Primers used in qRT-PCR are listed in Table S7. All experiments were repeated three times.

*Public gene expression database*

A total of 451 patients with PTC with known LNM status and 59 normal tissue adjacent to tumour (NAT) samples that were paired with the tumour tissues were also retrieved for further analysis. The clinical, genomic, and transcriptomic data of primary cancers were obtained from the TCGA data portal via the University of California Santa-Cruz (UCSC) Cancer Genome Browser (Accession date: 8 January 2022).^10^ To assess the transcriptomics, raw counts were normalised by the method used for DEG analysis and then log 2 transformed, and finally, normalisation into z-score was applied.^11^

Likewise, we retrieved clinical and gene expression data of 405 breast cancer samples with LNM, 26 melanoma samples with no LNM or only regional metastasis, and 95 lymph node metastasis samples of melanoma from TCGA (Accession date: 14 September 2022). We applied the same procedure and analysis for all TCGA sample data.

A total of 17 paired gene expression profiles (Affymetrix Human Genome U133 Plus 2.0) of PTC nodal metastases associated with primary tumours were obtained from the GSE60542 dataset.^12^ Raw intensity expression values were processed and normalised, as described in a previous study^12^. Expression of genes with multiple probes analysed through the Affymetrix platform were calculated by averaging all designated probes. Likewise, seven paired gene expression profiles (Affymetrix Human Genome U133 Plus 2.0) of PTC invaded areas associated with primary tumours were obtained from the GSE6004 dataset.^13^ The mRNA expression profiles (Thermo Fisher Human Clariom S Assay) of 34 PTC samples (17 primary PTCs and 17 radioactive iodine therapy resistant LNMs) were collected from GSE151179.^14^ Lastly, mRNA expression profiles (Affymetrix Human Gene Expression Array) of three non-aggressive PTC patients and three invasive patients were enrolled from GSE129880.^15^

Lymph node metastasis samples of other tumour types were collected from GSE56493 (GPL10379 Rosetta/Merck Human RSTA custom Affymetrix 2.0) and GSE65904 (GPL10558, Illumina Human HT-12).^16,17^ Forty-four lymph node metastasis of breast cancer and 130 lymph node metastasis of melanoma patient samples with both clinical information and gene expression data were obtained, and processing was performed as previously described.

Patient gene expression datasets for 20 anaplastic thyroid cancers and 17 poorly differentiated thyroid cancers were collected from GSE76039 for validation of LASSO score analysis (Affymetrix Human Genome U133 Plus 2.0).^18^

*Gene signature development*

Differential expression workflow for RNA-seq data based on the DESeq2 package from the Bioconductor package (<https://bioconductor.org/packages/release/bioc/html/DESeq.html>) was applied to define DEGs between LNM (−) and LNM (+) tumour samples from our in-house PTC cohort.^11^ Significant DEGs, those with p value below 0.05 adjusted using the Bonferroni correction method and FDR q value below 0.1, were selected.

*Machine learning algorithms and risk score construction*

To discover potential biomarkers related with LNM, least absolute shrinkage and selection operator (LASSO) regression analysis was used. The ratio of training set to test set was 7:3. Next, GridSearchCV was used to find the optimal hyper parameters from the model, and five-fold cross validation was applied with an alpha of 0.08. Further, the extreme gradient boosting algorithm (XGBOOST) was applied to confirm the model’s performance.^19^ SHAP (Shapley additive explanations), a value that evaluates the magnitude of feature attribution, was used to explain the individual prediction results in the XGBOOST model.^20^ Risk scores of the gene signature model were calculated according to relative abundance (Exp) and LASSO regression coefficients (β) of the genes selected as important features (LASSO-based risk score = each gene ∑ [β(i) × Exp(i)]). Using LASSO-based risk scores, patient samples higher or lower than 0.5 were assigned to different labels. For data that did not fully cover the proposed gene lists for model construction, expression levels of missing genes were imputed with a value of 0 after normalization. Every machine learning algorithm and the related visualisation plots were processed with the sci-kit learn packages of Python programming.

*K-means clustering algorithm*

The K-means algorithm was selected for unsupervised clustering to partition patient samples into groups of observations with the nearest mean to input variables. Clustering was performed with DEGs found in previous analyses, and this was conducted using sci-kit learn packages. To assess the accuracy of the clustering technique, silhouette scores were calculated for each k (k=2, 3, 4, 5, and 6). Silhouette scores ranged from −1 to 1, where scores closer to 1 indicated that clusters were well apart from each other.

*Functional enrichment analysis*

DEGs were analysed using g:Profiler with GO:MF, GO:BP, GO:CC, KEGG, REACTOME, and TRANSFAC databases.^6^ The enriched gene sets were selected with FDR < 0.05 corrected by the g:SCS algorithm in every database. Significant gene sets from GO:MF, GO:BP, and GO:CC were collectively assessed for clustering using the algorithm provided by DAVID: functional annotation tools.^21^ Functional clusters with enrichment scores greater than 2.0 were considered significant.

*Gene expression analysis*

Single sample GSEA (ssGSEA) v10.0.9 of GenePattern (<https://www.genepattern.org/>) was used to calculate separate enrichment scores for each pairing of a sample and gene set.^22^ Enrichment scores gained from ssGSEA represented the degree of up- or down-regulation of a specific gene set within a sample. Gene lists used for ssGSEA to address enrichment scores of thyroid differentiation, BRAF/RAS mutation score, and ERK score were referred from previous TCGA work.^23^ Specific gene lists are listed in Supplementary Table 2. EMT (Hallmark Epithelial Mesenchymal Transition) scores were calculated with an established gene matrix file registered in MSigDB.^24-26^

The xCell algorithm, a cell type enrichment analysis tool, was used to interpretate the tumour microenvironment with an immune score, stromal score and microenvironment score. Input data proceeded according to a guided protocol.^27^

*General Statistical analysis*

Normalised counts were log 2 transformed before gene expression analysis. Then, gene expressions and enrichment scores were normalised to z-scores within each cohort. For general statistics, paired t-test and unpaired t-test with Welch’s correction (two-sided) were performed with a 95% CI for continuous variable. Further, the chi-square test or Fisher’s exact test was chosen for categorical variables depending on analysed sample number. Correlation analysis was applied using Pearson’s correlation coefficients. Furthermore, progression free survival (PFS) was determined using Kaplan‒Meier survival curves, and significance values were generated via the log-rank test. P-values less than 0.05 were considered significant in all analyses. Multivariate analysis was performed to discover potential clinical indicators of risk scores. Reference values for categorical variables were chosen and compared by classes that represent normal or benign.

*Visualisation tools*

Heatmaps were constructed to identify DEGs, and hierarchical clustering of DEGs was performed with Morpheus software (https://software.broadinstitute.org/morpheus).^28^ Visualised plots (e.g., bar charts, logistic regression, heatmap, correlation, volcano plot) were generated using GraphPad Prism 9 (GraphPad Software Inc., San Diego, CA, USA).

**Table S1. Baseline characteristics of our in-house cohort and the TCGA THCA cohort**

|  | | **In-house cohort**  **(N = 292, %)** | **TCGA**  **(N = 451, %)** | **p value**  (χ^2^ test, t-test) |
| --- | --- | --- | --- | --- |
| Gender | Male | 74 (25.3) | 124 (27.5) | 0.5170 |
|  | Female | 218 (74.7) | 327 (72.5) |  |
| Lymph node metastasis^††^ | N0 | 97 (33.2) | 226 (50.1) | <0.0001*** |
|  | N1 | 195 (66.8) | 225 (49.9) | 0.0253* |
|  | N1a | 85 (29.1) | 93 (20.7) |  |
|  | N1b | 110 (37.7) | 75 (16.6) |  |
| Age |  | 50.5  (std = 14.2) | 47.2  (std = 15.6) | 0.8280 |
| *BRAF^V600E^* mutation | Absence | 41 (14.0) | 231 (51.1) | <0.001*** |
|  | Presence | 251 (86.0) | 220 (48.8) |  |
| Fusion genes | RET | 13 (4.5%) | 24 (5.3%) | 0.0079** |
|  | NTRK | 4 (1.3%) | 11 (2.4%) |  |
|  | Others^†††^ | 0 (0.0%) | 10 (2.2%) |  |
| T stage | T1 | 53 (18.2) | 131 (29.0) | <0.001*** |
|  | T2 | 18 (6.1) | 141 (31.2) |  |
|  | T3 | 199 (68.1) | 156 (34.6) |  |
|  | T4 | 31 (10.6) | 22 (4.9) |  |
|  | TX | 0 | 1 (0.2) |  |
| Extrathyroidal extension^††^ | None | 61 (20.9) | 290 (64.3) | <0.001*** |
|  | Minimal (Yes) | 231 (79.1) | 126 (27.9) |  |
|  | Moderate/Advanced | - | 18 (3.9) |  |
| Recurrence/Progression^†^ |  | 5 (0.02%) | 46 (10.2%) | <0.001*** |

**p* < 0.05, ***p* < 0.01, ****p* < 0.001

^†^ Clinical analysis with our in-house cohort was not appropriate due to the short follow-up period (minimum follow up period was 13 months).

^††^ Number of patients analysed does not equal the total number of TCGA patients since clinical information for a few patients was invalid: information for lymph node metastasis and extrathyroidal extension was missing in 54 and 15 patients, respectively.

^†††^ The fusion genes analysed were as follows: EML4::ALK, FGFR2::OFD1, GTF2IRD1::ALK, MET::TFG, PAX8::PPARG, STRN::ALK, UACA::LTK, and VCL::FGFR2.

^∫^ T-, N-, M-, TNM- stage according to the AJCC TNM staging system 7^e^

**Table S2.** List of genes used to calculate thyroid differentiation score (TDS) and BRAF, RAS, and ERK scores independently.

| Score Name | Gene Name |
| --- | --- |
| TDS | DIO1, DIO2, DUOX1, DUOX2, FOXE1, GLIS3, TTF1, PAX8, SLC26A4, SLC5A5, SLC5A8, TG, THRA, THRB, TPO,TSHR |
| BRAF | ABTB2, FLJ23867, PROS1, AHR, FN1, PTPRE, ANKLE2, FSTL3, NECTIN4, KCNIP3, GABRB2, RASGEF1B, ANXA1, GBP2, RUNX1, ANXA2P2, ITGA3, RUNX2, ARNTL, ITGB8, SDC4, ASAP2, KCNN4, SEL1L3, BID, LAMB3, SFTPB, CDC42EP1, LLGL1, SLC35F2, COL8A2, LY6E, SOX4, CREB5, MDFIC, SPOCK2, CTSC, MET, STK17B, CYP1B1, PDE5A, SYT12, DTX4, PDLIM4, TACSTD2, DUSP5, PLCD3, TBC1D2, ETHE1, PLEKHA5, TGFBR1, EVA1A, PNPLA5, DCSTAMP, FAM20C, PPL, TMEM43, FCHO1, PRICKLE1 |
| RAS | ANKRD4 CYB561, GNA14, HGD, KATNAL2, KCNAB1, LGI3, MLEC, NQO1, SFTPC, SLC4A4, SORBS2 |
| ERK | DUSP6, PLK3, MAFF, LIF, FOSL1, IL8, IER3, TNFRSF12A, PHLDA2, TNC, SLC1A5, ETV4, ETV5, ETV1, CCND1, DUSP4, HMGA2, SPRED2, CD3EAP, HYDIN, B4GALT6, SLC4A7, CHSY1, ELOVL6, ARID5A, KIR3DL2, GTF2A1L, MYC, YRDC, GNL3, BRIX1, RRS1, DDX21, WDR3, TSR1, GTPBP4, PPAT, GEMIN4, POLR3G, SPRY2, EGR1, FOS, GPR3, SH2B3, SPRY4, SEMA6A, MAP2K3, BYSL, PPAN, NOP16, POLR1C, PYCRL |

Gene matrix file used to calculate epithelial mesenchymal transition is registered in open molecular signature database as: HALLMARK_EPITHELIAL_MESENCHYMAL_TRANSITION.

**Table S3. Clinico-pathological characteristics according to LASSO score in our cohort and TCGA**

|  | | **Our cohort (n=97)** | | | **TCGA**  **(150)** | | | | **p value**  (χ^2^ test, t-test) | | | |  |  |
| --- | --- | --- | --- | --- | --- | --- | --- | --- | --- | --- | --- | --- | --- | --- |
|  |  | **Lower third** | **Upper third** | | | **Lower third** | **Upper third** | | | **Our cohort** | **TCGA** | | |  |
| Gender | Male | 34  (35.0%) | 21  (21.6%) | 43  (28.7%) | | | | 46  (30.6%) | | 0.038* | | 0.704 | | |
|  | Female | 63  (65.0%) | 76  (78.4%) | 107  (72.3%) | | | | 104  (69.4%) | |  |  |  |  |  |
| Lymph node metastasis^†^ | N0 | 49  (50.5%) | 18  (18.6%) | 109  (72.7%) | | | | 52  (34.7%) | | <0.001*** | | <0.001*** | | |
|  | N1 | 48  (49.5%) | 79  (81.4%) | 41  (27.3%) | | | | 98  (65.3%) | |  | |  | | |
|  | N1a | 25  (25.8%) | 34  (35.0%) | 15  (10%) | | | | 40  (26.7%) | |  | |  | | |
|  | N1b | 23  (23.7%) | 45  (46.4%) | 14  (9.3%) | | | | 33  (22%) | |  | |  | | |
| Age |  | 50.59 | 51.31 | 48.5 | | | | 50.2 | | 0.713 | | 0.347 | | |
| Tumour size |  | 1.9 | 1.81 | - | | | | - | | 0.643 | |  | | |
| *BRAF^V600E^* mutation | Absence | 23  (23.7%) | 5  (5.2%) | 124  (82.7%) | | | | 39  (26%) | | <0.001*** | | <0.001*** | | |
|  | Presence | 74  (76.3%) | 92  (94.8%) | 26  (27.3%) | | | | 111  (74%) | |  | |  | | |
| Fusion gene | RET | 2  (2.0%) | 3  (3.1%) | 4  (2.7%) | | | | 7  (4.6%) | | 0.5472 | | 0.9128 | | |
|  | NTRK | 3  (3.1%) | 1  (1.0%) | 5  (3.3%) | | | | 1  (0.6%) | |  | |  | | |
|  | Others^††^ | 0  (0.0%) | 0  (0.0%) | 2  (1.3%) | | | | 4  (2.7%) | |  | |  | | |
| T stage | T1 | 27  (27.8%) | 8  (8.2%) | 53  (35.3%) | | | | 35  (23.3%) | | <0.001*** | | 0.002** | | |
|  | T2 | 6  (6.2%) | 1  (1.0%) | 54  (36%) | | | | 40  (26.7%) | |  | |  | | |
|  | T3 | 55  (56.0%) | 73  (75.2%) | 39  (26%) | | | | 63  (42%) | |  | |  | | |
|  | T4 | 9  (9.3%) | 15  (15.4%) | 4  (2.6%) | | | | 11  (7.3%) | |  | |  | | |
|  | TX | 0 | 0 | 0 | | | | 1  (0.7%) | |  | |  | | |
| Extrathyroidal extension^†^ | None | 33  (34.0%) | 9  (9.3%) | 115  (76.7%) | | | | 76  (50.7%) | | <0.001*** | | <0.001*** | | |
|  | Minimal (Yes) | 64  (66%) | 88  (90.7%) | 21  (14%) | | | | 61  (40.7%) | |  |  |  |  |  |
|  | Moderate/  Advanced | - | - | 3  (2%) | | | | 10  (6.7%) | |  |  |  |  |  |
| M stage  (Other than lymph node) |  | 1  (1.0%) | 1  (1.0%) | 3  (2.1%) | | | | 2  (1.4%) | | >0.999 | | 0.853 | | |

**p* < 0.05, ***p* < 0.01, ****p* < 0.001

^†^ Number of patients analysed does not equal to the total number of TCGA patients since clinical information was invalid for a few patients (54 patients did not have specific lymph node metastasis status and 15 patients had extrathyroidal extension information missing in the TCGA cohort).

^††^ The fusion genes analysed were as follows: EML4::ALK, FGFR2::OFD1, GTF2IRD1::ALK, MET::TFG, PAX8::PPARG, STRN::ALK, UACA::LTK, and VCL::FGFR2.

**Table S4. Clinicopathological data of patients with lymph node metastasis compared between Cluster 1 and Cluster 2.** Data were analyzed in our cohort and TCGA.

| **In-house cohort** |  | **Cluster 1 N1 (N=62)** | **Cluster 2 N1 (N=133)** | **p value**  **(χ^2^ test, t-test)** |
| --- | --- | --- | --- | --- |
| Gender | Male | 27 | 29 | 0.004** |
|  | Female | 35 | 104 |  |
| Tumour size (cm) |  | 2.059 | 1.831 | 0.329 |
| Age |  | 50.06 | 50.49 | 0.843 |
| BRAF^V600E^ mutation | Absence | 11 | 10 | 0.032 * |
|  | Presence | 51 | 132 |  |
| T stage | T1 | 10 | 15 | 0.202 |
|  | T2 | 3 | 1 |  |
|  | T3 | 42 | 100 |  |
|  | T4 | 7 | 17 |  |
|  | TX | 0 | 0 |  |
| Extrathyroidal extension | No | 13 | 15 | 0.071 |
|  | Yes | 49 | 118 |  |

| **TCGA** |  | **Cluster 1 N1 (N=49)** | **Cluster 2 N1 (N=175)** | **p value**  **(χ^2^ test,**  **t-test)** |
| --- | --- | --- | --- | --- |
| Gender | Male | 15 | 55 | 0.913 |
|  | Female | 34 | 120 |  |
| Age |  | 45.2 | 45.5 | 0.910 |
| BRAF^V600E^ mutation | Absence | 35 | 71 | <0.001*** |
|  | Presence | 14 | 104 |  |
| T stage | T1 | 12 | 32 | 0.004** |
|  | T2 | 18 | 48 |  |
|  | T3 | 16 | 79 |  |
|  | T4 | 3 | 15 |  |
|  | TX | 0 | 1 |  |
| Extrathyroidal extension | None | 34 | 89 | 0.083 |
|  | Minimal | 11 | 71 |  |
|  | Moderate/Advanced | 2 | 12 |  |
|  | Very advanced | 0 | 1 |  |

**Table S5. Clinicopathological data of patients with lateral cervical lymph node metastasis (N1b) compared between Cluster 1 and Cluster 2**. Data were analyzed in our cohort and TCGA.

| **In-house cohort** |  | **Cluster 1 N1b (N=30)** | **Cluster 2 N1b**  **(N=80)** | **p value**  **(χ^2^ test, t-test)** |
| --- | --- | --- | --- | --- |
| Gender | Male | 17 | 16 | <0.001*** |
|  | Female | 13 | 64 |  |
| Tumour size (cm) |  | 2.426 | 1.907 | 0.241 |
| Age |  | 47.6 | 48.1 | 0.87 |
| BRAF^V600E^ mutation | Absence | 6 | 7 | 0.106 |
|  | Presence | 24 | 73 |  |
| T stage | T1 | 1 | 9 | 0.47 |
|  | T2 | 1 | 1 |  |
|  | T3 | 23 | 61 |  |
|  | T4 | 5 | 9 |  |
|  | TX | 0 | 0 |  |
| Extrathyroidal extension | No | 2 | 9 | 0.475 |
|  | Yes | 28 | 71 |  |

| **TCGA** |  | **Cluster 1 N1b (N=16)** | **Cluster 2 N1b (N=58)** | **p value**  **(χ^2^ test,**  **t-test)** |
| --- | --- | --- | --- | --- |
| Gender | Male | 6 | 24 | 0.779 |
|  | Female | 10 | 34 |  |
| Age |  | 48.3 | 49.1 | 0.873 |
| BRAF^V600E^ mutation | Absence | 13 | 26 | 0.009** |
|  | Presence | 3 | 32 |  |
| T stage | T1 | 4 | 11 | 0.908 |
|  | T2 | 3 | 13 |  |
|  | T3 | 8 | 27 |  |
|  | T4 | 1 | 6 |  |
|  | TX | 0 | 1 |  |
| Extrathyroidal extension | None | 10 | 29 | 0.007** |
|  | Minimal | 5 | 5 |  |
|  | Moderate/Advanced | 1 | 24 |  |

**Table S6. Mutation landscape of patients with PTC in Cluster 1 and Cluster 2 from our in-house and TCGA cohorts.** BRAF (V600E), RAS (NRAS Q61R, HRAS Q61R, KRAS Q61R), and pTERT (C228T, C250T, C228A) mutations were evaluated. In our in-house cohort, only one RAS mutation was detected in 292 patients with PTC. ****p* < 0.001.

|  | Our cohort | | *p*–value (χ^2^ test) |
| --- | --- | --- | --- |
|  | Cluster 1  (n = 127) | Cluster 2  (n = 165) | Cluster 1 vs Cluster 2 |
| BRAF mutation | 96 (75.6%) | 153 (92.7%) | 0.0003^***^ |
| RAS mutation | 1 (0.08%) | 0 (0%) |  |
| pTERT mutation | 15 (11.8%) | 16 (9.7%) |  |
| BRAF, RAS, and pTERT mutation (-) | 29 (22.8%) | 11 (6.7%) |  |
| RET fusion gene | 5 (3.9%) | 8 (4.8%) | 0.2941 |
| NTRK fusion gene | 3 (2.4%) | 1 (0.6%) |  |

|  | TCGA THCA | | *p*–value (χ^2^ test) |
| --- | --- | --- | --- |
|  | Cluster 1  (n = 177) | Cluster 2  (n = 274) | Cluster 1 vs Cluster 2 |
| BRAF mutation | 40 (22.5%) | 203 (74.1%) | <0.0001^***^ |
| RAS mutation | 47 (26.6%) | 1 (0.0%) |  |
| pTERT mutation | 6 (4.7%) | 31 (11.3%) |  |
| BRAF, RAS, and pTERT mutation (-) | 84 (47.6%) | 39 (14.2%) |  |
| RET fusion gene | 8 (4.5%) | 19 (6.9%) | 0.3022 |
| NTRK fusion gene | 5 (2.8%) | 9 (3.3%) |  |
| Other fusion gene ^†^ | 4 (2.3%) | 6 (2.1%) |  |

^†^ The fusion genes analysed were as follows: EML4::ALK, FGFR2::OFD1, GTF2IRD1::ALK, MET::TFG, PAX8::PPARG, STRN::ALK, UACA::LTK, and VCL::FGFR2.

**Table S7.** List of primers used in this study.

| DNA work | | | | |
| --- | --- | --- | --- | --- |
| Target gene | | Forward primer | | Reverse primer |
| TERT promoter | | 5’-ACGAACGRGGCCAGCGGCAG-3’ | | 5’-CTGGCGTCCCTGCACCCTGG-3’ |
| BRAF V600E | | 5’-ATGCTTGCTCTGATAGGAAA-3’ | | 5’-ATTTTTGTGAATACTGGGAA-3’ |
| NRAS Q61R | | 5’- AGCATTGCATTCCCTGTGGT-3’ | | 5’- AGTGTGGTAACCTCATTTCCCC-3’ |
| HRAS Q61R | | 5’-TCTCAGCACCCCAGGAGAG-3’ | | 5’-GACATGCGCAGAGAGGACAG-3’ |
| KRAS G12V | | 5’-CACGTCTGCAGTCAACTGGA-3’ | | 5’-ACCCTGACATACTCCCAAGGA-3’ |
| KRAS Q61K | | 5’-CCGTCATCTTTGGAGCAGGA-3’ | | 5’-TCCACTGCTCTAATCCCCCA-3’ |
| RNA work | | | | |
| Target gene | | Forward primer | | Reverse primer |
| NOX4 | 5’-TGTGCCGAACACTCTTGGC-3’ | | 5’-ACATGCACGCCTGAGAAAATA-3’ | |
| TULP3 | 5’-TGAGCCATTTATGGTGCAGC-3’ | | 5’-GCAGTATCCACGGTGTTTTCAG-3’ | |
| IFNGR1 | 5’-AGCAGGAAGTCGATTATGATCCC-3’ | | 5’-CTGGCACTGAATCTCGTCACA-3’ | |
| CTSC | 5’-CCAACTGCACCTATCTTGACC-3’ | | 5’-AAGGCAAACCACTTGTAGTCATT-3’ | |
| RIN1 | 5’-TCCTCGTGCGGAAATCTAACA-3’ | | 5’-GTGTGGCAGTAGGCACAGA-3’ | |
| BIN1 | 5’-TGAGCAGTGCGTCCAGAATTT-3’ | | 5’-CGATCTTGTTTGCCTCATCCC-3’ | |
| SYBU | 5’-TTCTTCACGCAATCGAGGTCC-3’ | | 5’-GGGCTACAGTCGCTTCCTTT-3’ | |
| STARD7 | 5’-GAGATGAAGCGGTTGGAAGAA-3’ | | 5’-ACTCGGTACTGGTAAAGGTGG-3’ | |
| FAM53A | 5’-CTTTGCTCCCTCAATTACGAAGA-3’ | | 5’-CTGGTCACCGACTCCCTAGA-3’ | |
| GAMT | 5’-CGCCCATTGATGAGCATTGG-3’ | | 5’-GGCCTTTCAAGGGGATGACC-3’ | |

**Figure S1. Schematic summary of the key process.** Gene expression profiles were obtained from 292 patients with PTC in our in-house cohort using Illumina RNA sequencing (NovaSeq 6000 platform). DESEQ2 was conducted between N0 and N1 patient samples and LNM relevant DEGs were identified. For DEGs, K-means unsupervised clustering algorithm was used to identify two distinctive molecular subtypes. To assess these subtypes, a risk model was built using LASSO machine learning with important gene signatures found via GridSearchCV of the sci-kit learn software supported in Python. Validation of our gene signatures was done in terms of clinical outcomes and biological characteristics in public PTC datasets: 1) 451 primary tumours paired with 59 normal tissue adjacent to tumour samples of TCGA-THCA; 2) 17 paired primary PTC to LNM samples in GSE60542; and 3) 13 normal thyroid samples, 17 primary PTC, five synchronous LNM, and 17 radioactive iodide therapy refractory (RAI-R) LNM samples from GSE151179 were collected. Abbreviations: RAI, radioactive iodine; PTC, papillary thyroid cancer; LNM, lymph node metastasis; DEGs differentially expressed genes.


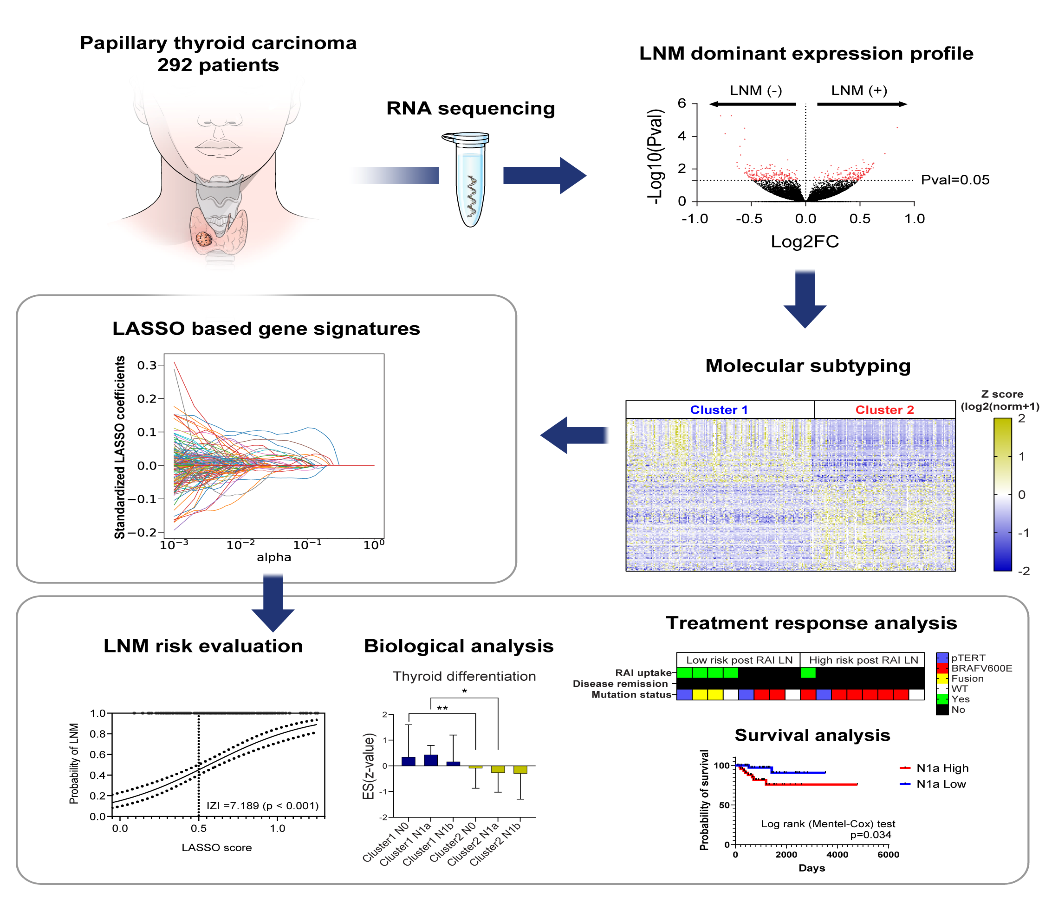

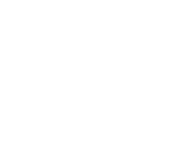

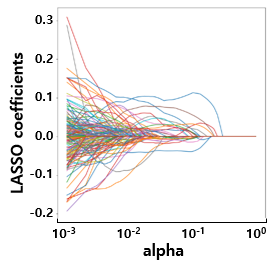


**Figure S2. Functional annotation analysis of DEGs found dominantly in patients with LNM and without LNM.** (A, B) Top 15 gene sets were marked with numbering, and specific profiles of the top 15 gene sets with p-values were charted in both LNM (+) and LNM (-) patient groups. (C) Functional annotation clustering of significant gene sets from GO: MF, BP, and CC using DAVID functional clustering. Two significant clusters with enrichment score (ES) values greater than 2.0 are presented.


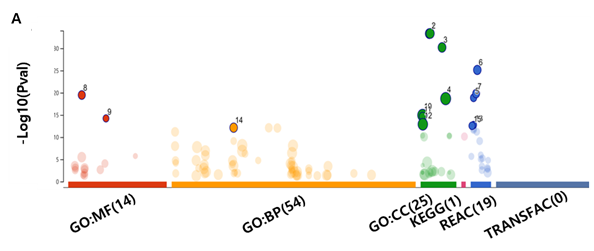


| **ID: Gene set*** | **P-VALUE** |
| --- | --- |
| GO:0030312: external encapsulating structure | 4.666*10^-34^ |
| GO:0031012: extracellular matrix | 4.325*10^-34^ |
| GO:0062023: collagen-containing extracellular matrix | 6.069*10^-31^ |
| REAC:R-HSA-1474244: extracellular matrix organization | 7.130*10^-26^ |
| GO:0005201: extracellular matrix structural constituent | 3.178*10^-20^ |
| REAC:R-HSA-1474228: degradation of the extracellular matrix | 1.473*10^-20^ |
| GO:0071944: cell periphery | 2.052*10^-19^ |
| REAC:R-HSA-1442490: collagen degradation | 1.335*10^-19^ |
| GO:0030020: extracellular matrix structural constituent conferring tensile strength | 5.834*10^-15^ |
| GO:0005581: collagen trimer | 1.929*10^-15^ |
| GO:0005576: extracellular region | 1.115*10^-15^ |
| GO:0030198: extracellular matrix organization | 7.144*10^-13^ |
| REAC:R-HSA-2022090: assembly of collagen fibrils and other multimeric structures | 2.670*10^-13^ |
| REAC:R-HSA-8948216: collagen chain trimerization | 1.760*10^-13^ |
| GO:0005615: extracellular space | 1.234*10^-13^ |

*Gene sets are ranked in decremental order. Abbreviations: LNM, lymph node metastasis.


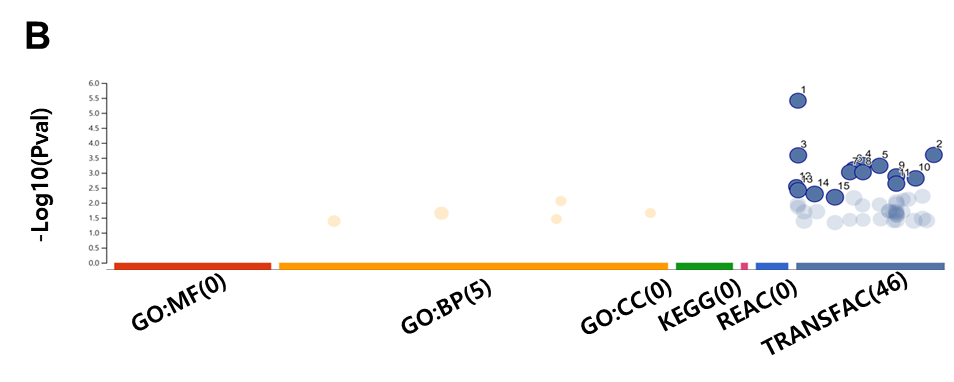


**B**

| **TF Name** | **P-VALUE** |
| --- | --- |
| TF: AP-2beta (M01858) | 3.891*10-6 |
| TF: ZNF615 (M09862) | 2.513*10-4 |
| TF: AP-2 (M00915) | 2.597*10-4 |
| TF: MOVO-B (M01104_1) | 5.336*10-4 |
| TF: PHB (M12725) | 5.795*10-4 |
| TF: Kaiso (M03876_1) | 7.578*10-4 |
| TF HTF4 (M02018) | 9.509*10-4 |
| TF: MRF4 (M03831) | 9.509*10-4 |
| TF: Sp1 (M00933) | 1.305*10-3 |
| TF: ZIC4 (M12227) | 1.520*10-3 |
| TF: Sp1 (M00932) | 2.283*10-3 |
| TF: AhR (M00976) | 2.874*10-3 |
| TF: AP-2 (M00800) | 3.778*10-3 |
| TF: E2F1 (M12597) | 5.060*10-3 |
| TF: GCMa:Erg (M08487) | 6.488*10-3 |

*Gene sets are ranked in decreasing order.

Abbreviations: TF, Transcription Factor.

**C**

**
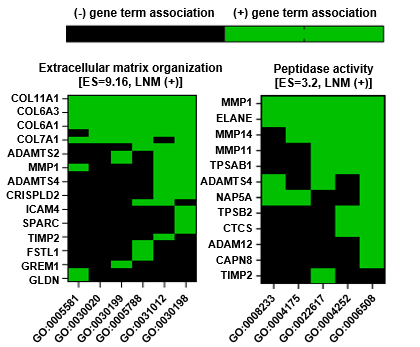
**

**Figure S3. Silhouette score coefficients for unsupervised k means clustering.** Assessment in 292 patients with PTC of our cohort with 244 DEGs via the sci-kit learn package. For K = 2, 3, 4, 5, and 6, the silhouette score coefficients were calculated as 0.736, 0.542, 0.544, 0.459, and 0.165, respectively.

**Figure S4. Full heatmap with expression values normalized within each gene (row).** Hierarchical clustering profiles of 244 DEGs (row) and K means cluster, LNM status, and BRAF mutation status information are provided (column). Visualization was performed using Morpheus.


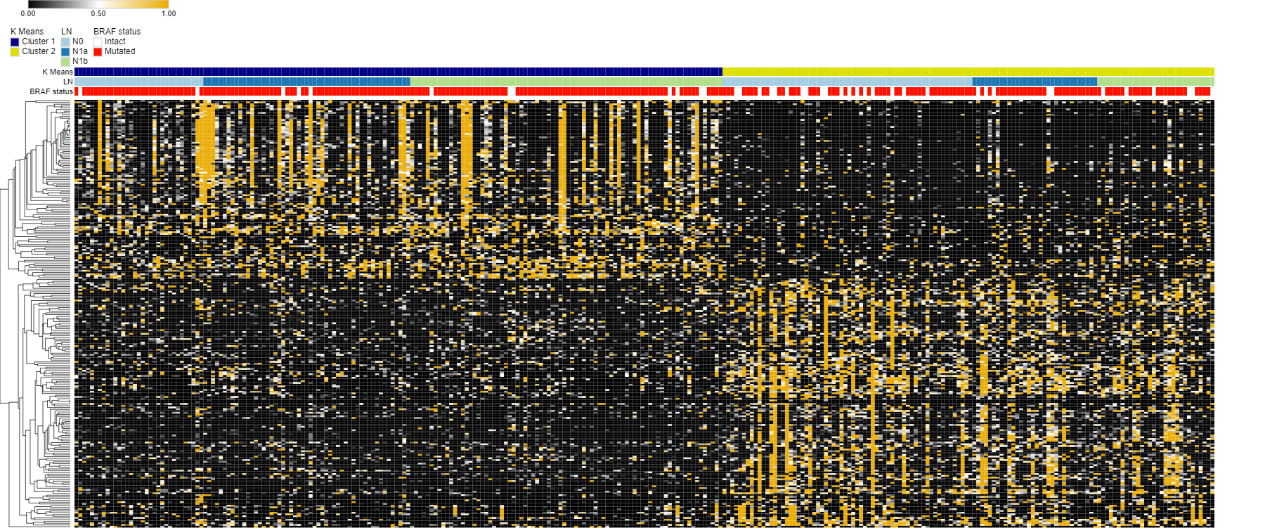


**Figure S5. Important gene signatures selected for machine learning model development. (A)** Name of genes and corresponding LASSO coefficient values selected as important gene signatures for risk prediction of Cluster 1 and Cluster 2. Genes are ordered in decremental order. Those positively predicting Cluster 2 (positive coefficient value) are represented in yellow and otherwise in blue. (**B**) Cross validation scores according to alpha regularization coefficient are depicted. A total of five cross validations were performed on corresponding models, and standard errors are shown as dotted lines. **(C)** Comparison of relative mRNA expression of 10 representative genes with the highest or lowest coefficients between 20 Cluster 1 and 20 Cluster 2 patient samples using real-time PCR. Patients were selected in their highest and lowest rank of order regarding LASSO score. P-values were calculated using the Mann-Whitney u test. *****p*<0.001.

**A**

| SELECTED FEATURE | |
| --- | --- |
| GENE | COEFFICIENT |
| NOX4 | 0.1004 |
| TULP3 | 0.063 |
| IFNGR1 | 0.0281 |
| CTSC | 0.0273 |
| RIN1 | 0.0269 |
| SLC22A4 | 0.024 |
| HRH1 | 0.0208 |
| COL10A1 | 0.0173 |
| TSC22D1 | 0.0165 |
| SEMA3A | 0.015 |
| RFX8 | 0.0062 |
| VAPB | -0.0016 |
| RPS6KA1 | -0.0058 |
| BIN1 | -0.007 |
| SYBU | -0.0106 |
| STARD7 | -0.0129 |
| FAM53A | -0.0282 |
| GAMT | -0.152 |


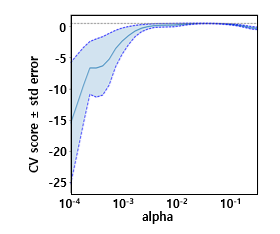


**B**

**C**


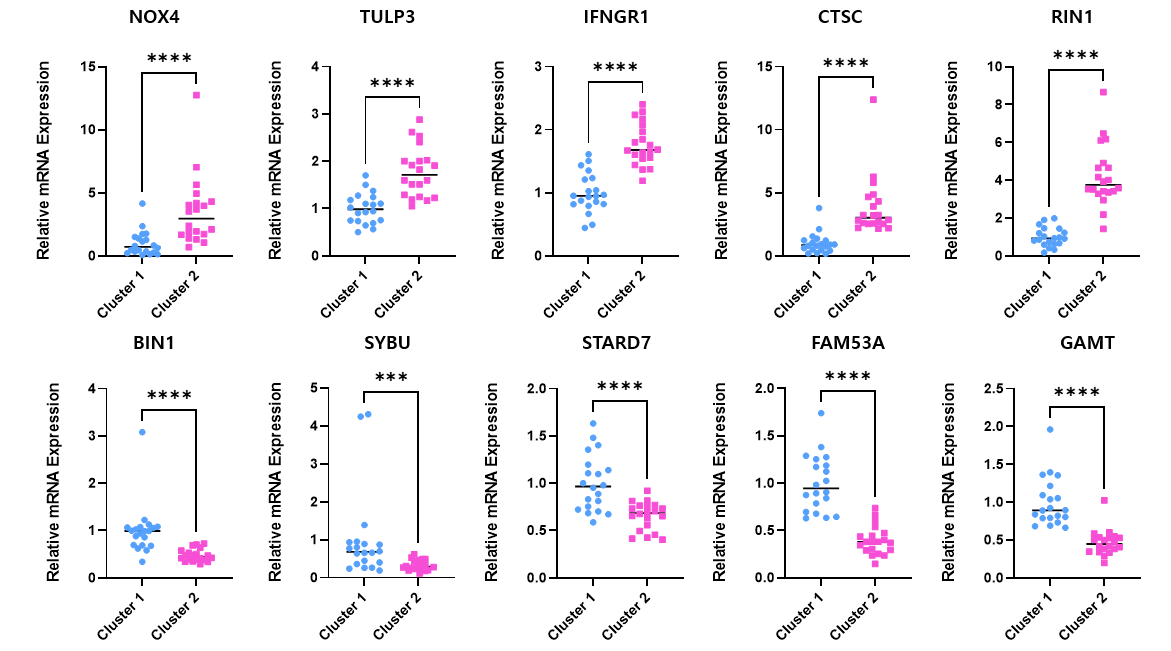


**Figure S6**. **Ensemble boosting algorithm to classify cluster 1 and cluster 2 in our in-house cohort. (A)** ROC curve (upper panel) and SHAP value summary plot (lower panel) from the extreme gradient boosting (XGBoost) algorithm conducted for PTC patients in our in-house cohort. **(B)** Feature importance plot for the XGBoost model, where the top 20 features are shown. The blue and red points in each row represent low to high values of the specific feature, while the *x*-axis shows the SHAP value, indicating the impact on the mode.


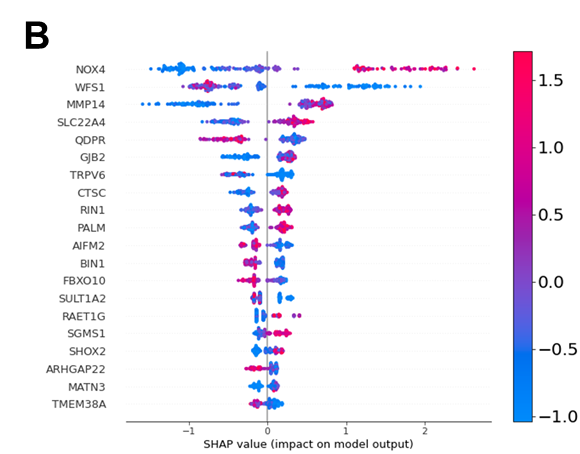


**B**


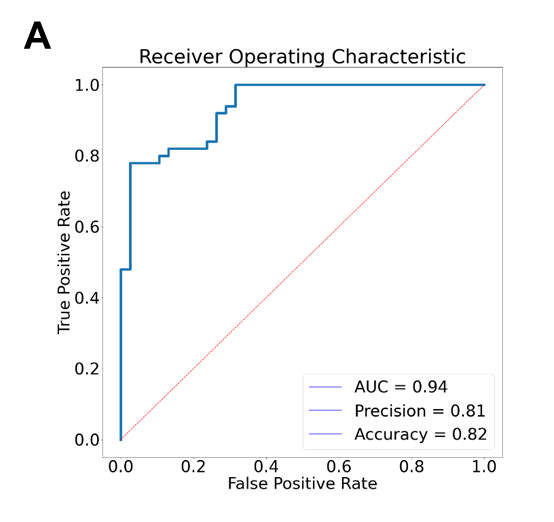


**A**

**Figure S7. Single sample GSEA analysis of HALLMARK pathways compared between Cluster 1 and Cluster 2 patient groups in the TCGA database.** A total of 20 significantly enriched hallmark gene sets with a *p*-value below 0.01 are graphed.

**Figure S8. Single sample GSEA analysis of HALLMARK pathways between LNM (-) and LNM (+) patient groups in each cohort.** HALLMARK pathways that differed significantly with *p*-values below 0.05 are listed for our cohort **(A)** and the TCGA database **(B)**, respectively.

**Figure S9. Number of cases of LNM (−), LNM (+), N1a, and N1b depicted as a bar graph in each cluster of the TCGA database.** Cluster 2 and Cluster 1 had an LNM risk correlated with an odds ratio of 4.691 (95% CI: 3.131-7.1).


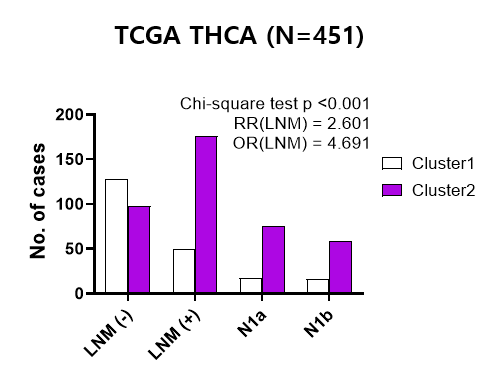


**Figure S10. Survival analysis of the progression free interval according to the status of LNM (A) and BRAFV600E mutation (B) in TCGA patients.** Statistics were computed using the log rank (Mantel‒Cox) test.

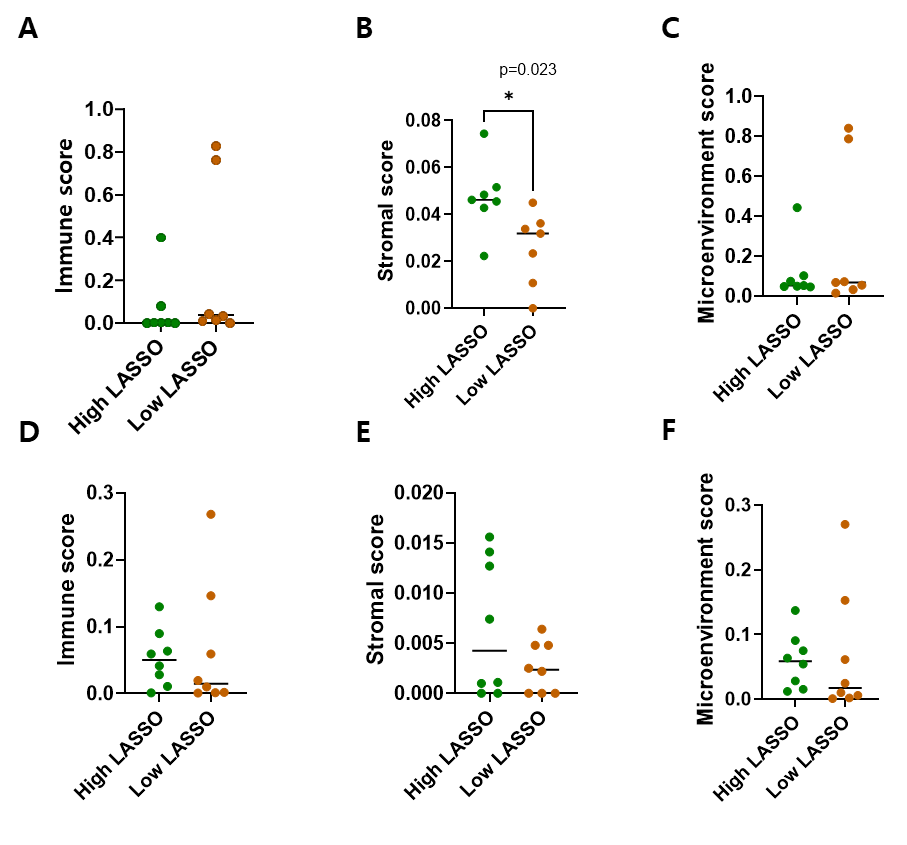
**Figure S11. Assessment of tumor microenvironment score using the xCell algorithm.** Immune score, stromal score and microenvironment score were calculated in lymph node metastasis samples of GSE60542 **(A-C)** and GSE151179 **(D-E)**. Calculation was done using the xCell algorithm, and unpaired t-test was utilized to evaluate statistical significance

|  |  |  |  |
| --- | --- | --- | --- |
|  |  |  |  |

**Figure S12. Proposed gene signatures are preserved throughout disease progression.** (A to C) LASSO scores were calculated for GSE60542, GSE6004, and TCGA data samples to compare score status of primary tumours to metastasis to that of paired invading tumour. (D) LASSO scores of all paired primary tumour and LNM samples were evaluated for correlation using Pearson correlation coefficients. (E) PTC samples of GSE129880 labelled with either indolent or invasive clinical outcomes were compared for LASSO scores, and significant differences were noticed. *p < 0.05. Abbreviations: PT, primary tumour; LNM, lymph node metastasis; TIA, tumour invaded area.


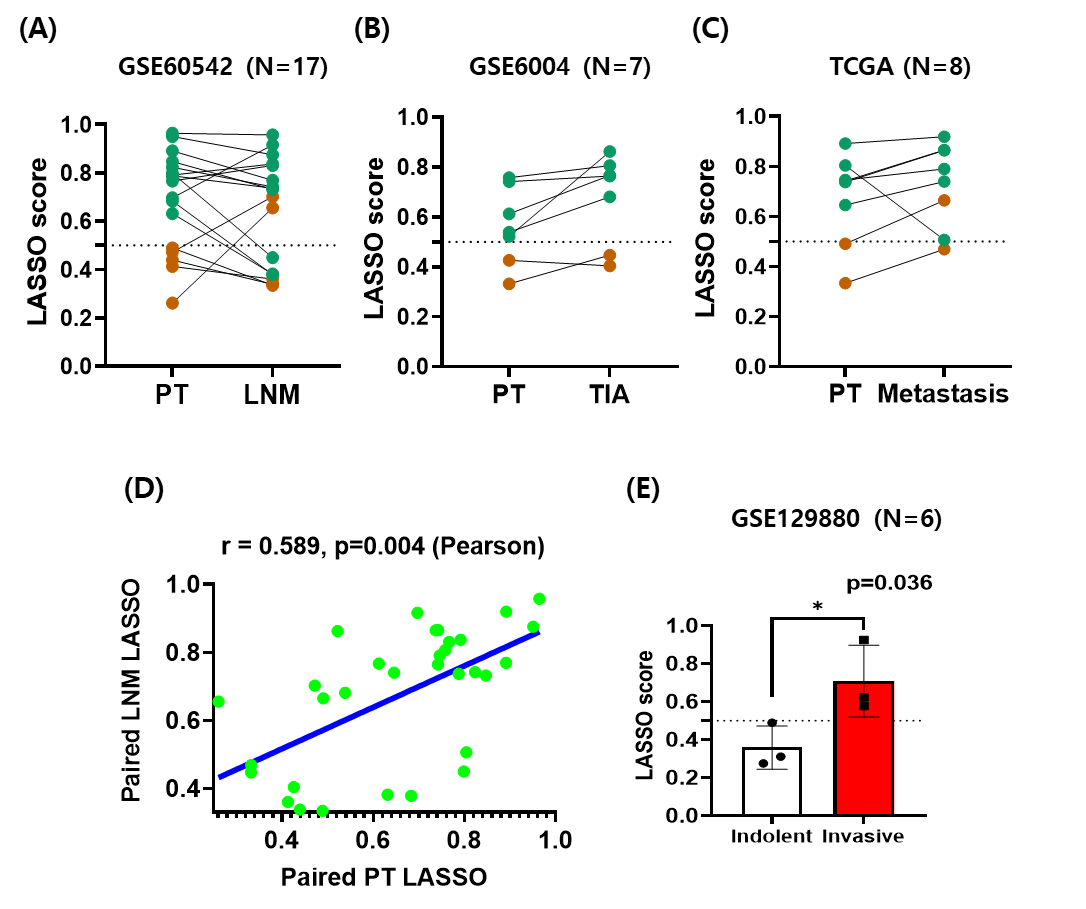


**A**

**B**

**C**

**D**

**E**

**Figure S13. Comparison of proposed LASSO scores between anaplastic thyroid cancer and poorly differentiated thyroid cancer. (A)** Gene expression profiles of 20 anaplastic thyroid cancers and 17 poorly differentiated thyroid cancers were analysed from GSE76039. LASSO scores between different tumour type were compared using unpaired t-test. **(B)** ssGSEA enrichment scores were compared between high and low LASSO score samples within each cancer cohort. Abbreviations: ATC, anaplastic thyroid cancer; PDTC, poorly differentiated thyroid cancer.

**A**


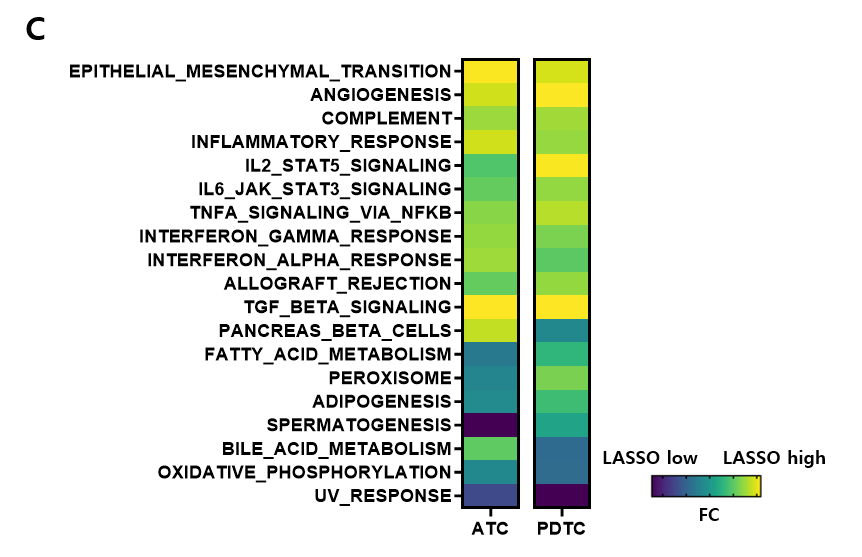


**B**

**Figure S14. Comparison of LASSO scores between patient samples with different radioactive iodine therapy responses and suppressed serum thyroglobulin levels at 1 year after treatment. (A)** The Mann Whitney U-test was conducted between patients who underwent none/low-dose RAIT and intermediate/high-dose RAIT. **(B)** Additionally, patient groups with no RAIT and high-dose RAIT were compared. **(C)** LASSO scores were compared according to suppressed serum Tg levels at 1 year after treatment in patients with high-dose RAIT. **p* < 0.05, ****p* <0.001. RAIT, radioactive iodine therapy; Tg, thyroglobulin.

**C**

**B**

**A**


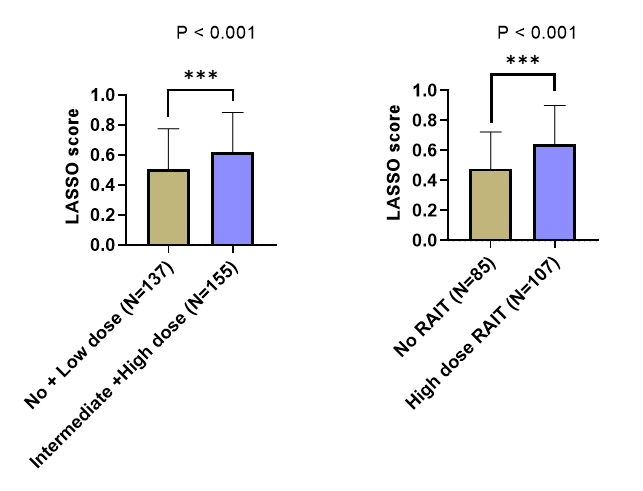


**Supplementary References**

1. Haugen BR, Alexander EK, Bible KC, et al. 2015 American Thyroid Association Management Guidelines for Adult Patients with Thyroid Nodules and Differentiated Thyroid Cancer: The American Thyroid Association Guidelines Task Force on Thyroid Nodules and Differentiated Thyroid Cancer. *Thyroid.* 2016;26(1):1-133.

2. Anatomic ACTAoDo, Surgical P. ADASP recommendations for processing and reporting of lymph node specimens submitted for evaluation of metastatic disease. *Mod Pathol.* 2001;14(6):629-632.

3. Randolph GW, Duh QY, Heller KS, et al. The prognostic significance of nodal metastases from papillary thyroid carcinoma can be stratified based on the size and number of metastatic lymph nodes, as well as the presence of extranodal extension. *Thyroid.* 2012;22(11):1144-1152.

4. Urken ML, Mechanick JI, Sarlin J, Scherl S, Wenig BM. Pathologic reporting of lymph node metastases in differentiated thyroid cancer: a call to action for the College of American Pathologists. *Endocr Pathol.* 2014;25(3):214-218.

5. Pertea M, Pertea GM, Antonescu CM, Chang TC, Mendell JT, Salzberg SL. StringTie enables improved reconstruction of a transcriptome from RNA-seq reads. *Nat Biotechnol.* 2015;33(3):290-295.

6. Raudvere U, Kolberg L, Kuzmin I, et al. g:Profiler: a web server for functional enrichment analysis and conversions of gene lists (2019 update). *Nucleic Acids Res.* 2019;47(W1):W191-w198.

7. McPherson A, Hormozdiari F, Zayed A, et al. deFuse: an algorithm for gene fusion discovery in tumor RNA-Seq data. *PLoS Comput Biol.* 2011;7(5):e1001138.

8. Uhrig S, Ellermann J, Walther T, et al. Accurate and efficient detection of gene fusions from RNA sequencing data. *Genome Res.* 2021;31(3):448-460.

9. Nicorici D, Satalan M, Edgren H, et al. FusionCatcher - a tool for finding somatic fusion genes in paired-end RNA-sequencing data. bioRxiv; 2014.

10. Goldman MJ, Craft B, Hastie M, et al. Visualizing and interpreting cancer genomics data via the Xena platform. *Nat Biotechnol.* 2020;38(6):675-678.

11. Love MI, Huber W, Anders S. Moderated estimation of fold change and dispersion for RNA-seq data with DESeq2. *Genome Biol.* 2014;15(12):550.

12. Tarabichi M, Saiselet M, Trésallet C, et al. Revisiting the transcriptional analysis of primary tumours and associated nodal metastases with enhanced biological and statistical controls: application to thyroid cancer. *Br J Cancer.* 2015;112(10):1665-1674.

13. Vasko V, Espinosa AV, Scouten W, et al. Gene expression and functional evidence of epithelial-to-mesenchymal transition in papillary thyroid carcinoma invasion. *Proc Natl Acad Sci U S A.* 2007;104(8):2803-2808.

14. Colombo C, Minna E, Gargiuli C, et al. The molecular and gene/miRNA expression profiles of radioiodine resistant papillary thyroid cancer. *J Exp Clin Cancer Res.* 2020;39(1):245.

15. Akyay OZ, Gov E, Kenar H, et al. Mapping the Molecular Basis and Markers of Papillary Thyroid Carcinoma Progression and Metastasis Using Global Transcriptome and microRNA Profiling. *Omics.* 2020;24(3):148-159.

16. Tobin NP, Harrell JC, Lövrot J, et al. Molecular subtype and tumor characteristics of breast cancer metastases as assessed by gene expression significantly influence patient post-relapse survival. *Ann Oncol.* 2015;26(1):81-88.

17. Cirenajwis H, Ekedahl H, Lauss M, et al. Molecular stratification of metastatic melanoma using gene expression profiling: Prediction of survival outcome and benefit from molecular targeted therapy. *Oncotarget.* 2015;6(14):12297-12309.

18. Landa I, Ibrahimpasic T, Boucai L, et al. Genomic and transcriptomic hallmarks of poorly differentiated and anaplastic thyroid cancers. *J Clin Invest.* 2016;126(3):1052-1066.

19. Chen T, Guestrin C. XGBoost: A Scalable Tree Boosting System. *Kdd '16.* 2016:785–794.

20. Lundberg SM, Lee S-I. A unified approach to interpreting model predictions. *Advances in neural information processing systems.* 2017;30.

21. Huang da W, Sherman BT, Lempicki RA. Systematic and integrative analysis of large gene lists using DAVID bioinformatics resources. *Nat Protoc.* 2009;4(1):44-57.

22. Reich M, Liefeld T, Gould J, Lerner J, Tamayo P, Mesirov JP. GenePattern 2.0. *Nat Genet.* 2006;38(5):500-501.

23. Integrated genomic characterization of papillary thyroid carcinoma. *Cell.* 2014;159(3):676-690.

24. Mootha VK, Lindgren CM, Eriksson KF, et al. PGC-1alpha-responsive genes involved in oxidative phosphorylation are coordinately downregulated in human diabetes. *Nat Genet.* 2003;34(3):267-273.

25. Subramanian A, Tamayo P, Mootha VK, et al. Gene set enrichment analysis: a knowledge-based approach for interpreting genome-wide expression profiles. *Proc Natl Acad Sci U S A.* 2005;102(43):15545-15550.

26. Liberzon A, Subramanian A, Pinchback R, Thorvaldsdóttir H, Tamayo P, Mesirov JP. Molecular signatures database (MSigDB) 3.0. *Bioinformatics.* 2011;27(12):1739-1740.

27. Aran D, Hu Z, Butte AJ. xCell: digitally portraying the tissue cellular heterogeneity landscape. *Genome Biol.* 2017;18(1):220.

28. . <https://software.broadinstitute.org/morpheus>.
